# An integrated proteomics approach shows synaptic plasticity changes in an APP/PS1 Alzheimer's mouse model

**DOI:** 10.18632/oncotarget.9092

**Published:** 2016-04-28

**Authors:** Stefan J. Kempf, Athanasios Metaxas, María Ibáñez-Vea, Sultan Darvesh, Bente Finsen, Martin R. Larsen

**Affiliations:** ^1^ Department of Biochemistry and Molecular Biology, University of Southern Denmark, Odense M, Denmark; ^2^ Institute of Molecular Medicine, University of Southern Denmark, Odense C, Denmark; ^3^ Department of Medical Neuroscience, Dalhousie University, Halifax, NS, Canada; ^4^ Department of Medicine (Neurology and Geriatric Medicine), Dalhousie University, Halifax, NS, Canada

**Keywords:** synapse, neuroinflammation, proteomics, miRNA, tau protein, Pathology Section

## Abstract

The aim of this study was to elucidate the molecular signature of Alzheimer's disease-associated amyloid pathology.

We used the double APP_swe_/PS1_ΔE9_ mouse, a widely used model of cerebral amyloidosis, to compare changes in proteome, including global phosphorylation and sialylated N-linked glycosylation patterns, pathway-focused transcriptome and neurological disease-associated miRNAome with age-matched controls in neocortex, hippocampus, olfactory bulb and brainstem. We report that signalling pathways related to synaptic functions associated with dendritic spine morphology, neurite outgrowth, long-term potentiation, CREB signalling and cytoskeletal dynamics were altered in 12 month old APP_swe_/PS1_ΔE9_ mice, particularly in the neocortex and olfactory bulb. This was associated with cerebral amyloidosis as well as formation of argyrophilic tangle-like structures and microglial clustering in all brain regions, except for brainstem. These responses may be epigenetically modulated by the interaction with a number of miRNAs regulating spine restructuring, Aβ expression and neuroinflammation.

We suggest that these changes could be associated with development of cognitive dysfunction in early disease states in patients with Alzheimer's disease.

## INTRODUCTION

Alzheimer's disease (AD) is the leading cause of dementia affecting more than 35 million people worldwide. Thus, it is crucial to obtain a better understanding of the molecular mechanisms underlying the neuropathology of AD to identify means to treat this disease.

Studies of human post-mortem brains and AD models indicate that synapses are affected at the earliest stages of neurodegenerative processes [1]. Importantly, AD is characterized by the accumulation of amyloid β-peptide (Aβ) in extracellular plaques and intracellular neurofibrillary tangles (NFTs), composed of hyper-phosphorylated microtubule-associated tau protein (Mapt), contributing to synaptic degeneration [2]. AD is attendant with a number of signs and symptoms including abnormalities in cognition, behaviour, olfaction, sleep wake cycle and autonomic dysfunction. These functions are subserved by a number of brain regions including neocortex, hippocampal formation, olfactory system and brainstem structures, playing a role in AD pathology [3] [4]. The development of Aß and tau pathology is predominantly observed in brain areas associated with cognition and memory, such as the neocortex and hippocampus [5], while several other regions, including the brainstem and olfactory bulb, seem to be relatively less affected and even understudied [5]. However, these brain regions are intertwined via the neocortex as well as hippocampal formation and contribute in that way to learning and cognition functions.

Generally, synaptic activity is one of the most important factors that regulate Aβ levels [6] and involves signalling mechanisms that shape signalling pathways of long-term potentiation (LTP) and –depression (LTD); modulation of these have been summarized as synaptic plasticity. While LTP is, in part, due to activation of protein kinases, such as calmodulin-kinases (Camk's), protein kinases-A (PKA`s) and -C (PKC`s), phosphorylating downstream target proteins, LTD arises from activation of calcium-dependent phosphatases such as types 1 (PP1) and 2 (PP2) dephosphorylating proteins [7]. Consequently, protein phosphorylation and -dephosphorylation are crucial events in regulating synaptic plasticity. Moreover, there is increasing evidence that N-linked glycosylation of proteins can also influence neural cell adhesion, axonal targeting and neuronal transmission (neurotransmitter release, -reception and -uptake) that also significantly contributes to pathogenesis of AD [8]. An abnormal glycosylation pattern can also influence chronic neuroinflammation [9] that is a hallmark in AD. Neuroinflammatory signalling can, in turn, be modulated by microRNA's (miRNA's) in AD [10]. A subset of miRNA's regulating both neuronal and immune processes (neurimmiRs) appear to be appropriate diagnostic biomarkers and therapeutic targets for neurodegenerative disorders, AD in particular [11]. Neuronal signalling pathways that modulate synaptic plasticity are multifactorial and include regulatory mechanisms involving expression modulation of mRNA, miRNA, protein and post-translational modification (PTM) of protein. Consequently, a simultaneous cross-molecular level investigation in multiple brain regions will further our understanding aboutthe neuropathology of AD.

Recently, we showed that the widely used APP_swe_/PS1_ΔE9_ (APP/PS1) double transgenic mouse model of AD, like patients with AD, show an age-dependent sigmoidal increase in cortical Aβ plaque load and a chronic neuroinflammatory reaction becoming prominent around 12 months of age [12], at a time point when the transgenic mouse shows cognitive and behavioural deficits [13]. We therefore used this time point for our integrated -omics approach to gain more insight into the molecular neuropathology of AD in several brain regions to study signalling pathways that regulate synaptic plasticity. These APP_swe_/PS1_ΔE9_ mice were originally made by co-injecting two vectors encoding mutant APP (K670N/M671L) and mutant PSEN1 (PSEN1ΔE9). These mice begin to develop Aβ depositis by 6 months of age, with abundant plaques in the neocortex and hippocampus by 12 months and the Aβ load increases up till 18 months of age whereafter it plateaus [12]. Formation of NFTs is not typical in these transgenic mice.

In the present work, we show that the olfactory bulb, neocortex, brainstem and hippocampus have a distinctly different molecular signature on the synaptic plasticity-mediated signalling pathways in male APP_swe_/PS1_ΔE9_ mice at an age of 12 months compared to the age-matched controls. The hippocampus seems to be less affected in our analysis. Stathmin-mediated microtubuli processing and the formation of argyrophilic tangle-like structures as well as CREB-mediated synaptic signalling represent the major changes observed, particularly in the neocortex and olfactory bulb.

## RESULTS

### The APP/PS1 mutation leads to changes in cytoskeletal proteins associated to synapses

The protein quantification showed a substantial increase in the number of deregulated unmodified proteins in the olfactory bulb (100 proteins) whereas only a few proteins were affected by the APP/PS1 mutation in the hippocampus (6 proteins), brainstem (12 proteins) and neocortex (4 proteins) (Figure 2A). There was no single altered protein overlapping between these different brain regions. Phospho- and glyco-proteomics revealed a similar picture, where the olfactory bulb showed most changes in PTMs (127 proteins) compared to the lower number in the hippocampus (39 proteins), brainstem (115 proteins) and neocortex (53 proteins) (Figure 2B). This is based on the number of phosphorylated / formerly sialylated N-linked glycosylated peptides found to be changed in the olfactory bulb (203), hippocampus (52), brainstem (199) and neocortex (72) (Figure 2C). A PCA analysis of all quantifiable peptides depicts this context (Figure 2F and 2G). There was a distinct grouping of the wild-type and APP/PS1-mutated tri-replicates in each brain region, respectively, whereas the greatest distance, meaning biological difference, was noted in the olfactory bulb (Figure 2F and 2G). Using information from PhosphoSitePlus database related to AD, we noted that Mapt-Ser506, Stmn1-Ser25 and Stmn1-Ser38, that are altered in the olfactory bulb of our study, are known phosphosites in AD. However, we found also a number of new sites related to phosphorylation in these proteins and others (SI Tables S2–S5). The complete list of deregulated proteins and formerly sialylated N-linked-glycosylated as well as phosphorylated proteins with corresponding peptide sequences and PANTHER protein class annotation can be found in Supplementary Information (SI Tables S1–S5).

**Figure 1:**
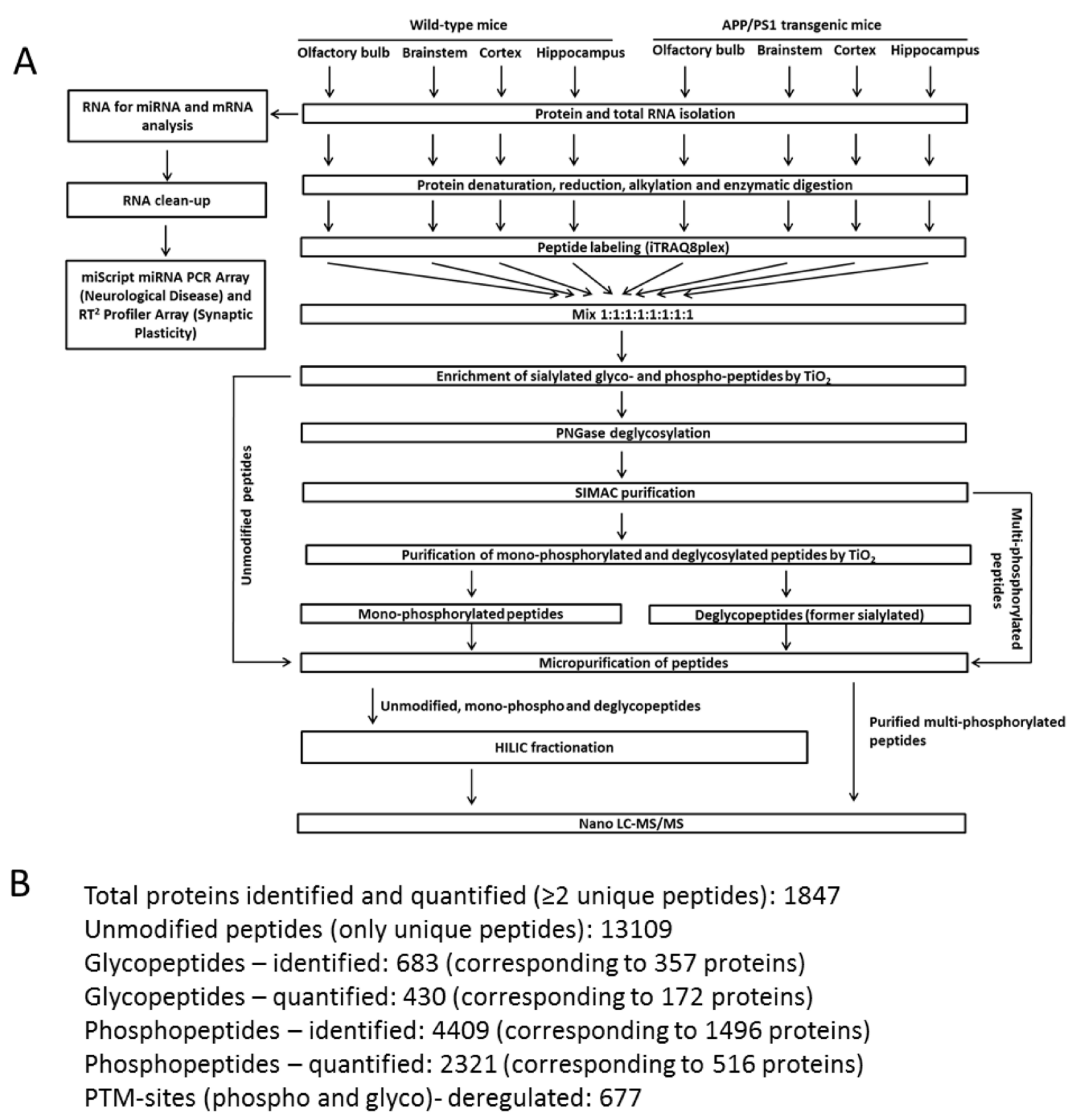
Sample processing workflow for miRNA, mRNA and proteome quantification Panel A shows the complete workflow. Panel B highlights the total number of proteins, post-translational modified phospho-/glyoc-peptides and proteins. HILIC, hydrophilic interaction chromatography; LC-MS/MS, liquid chromatography-tandem mass spectrometry.

**Figure 2:**
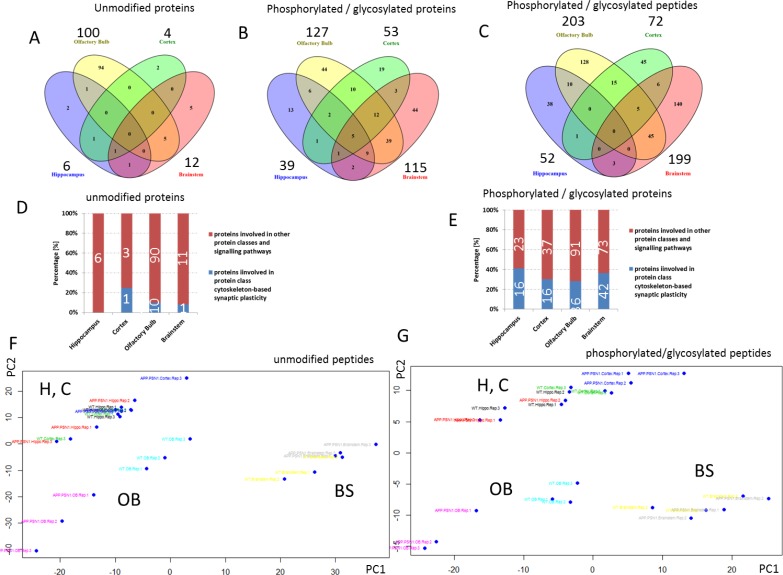
Mass spectrometry-based proteomics of proteins and post-translational modified proteins Venn diagram showing the number of all and shared deregulated unmodified proteins **A.** and phosphorylated / N-linked glycosylated proteins **B.** as well as peptides **C.** in the hippocampus, olfactory bulb, neocortex and brainstem in 12 month old APP/PS1 male mice compared against age-matched controls; *n* = 3. The number above each brain region shows the total number of changed hits per brain region. The number of deregulated hits from the global proteomics experiments in the hippocampus, neocortex, olfactory bulb and brainstem belonging to the protein class “cytoskeleton-based synaptic plasticity” (Table S1–S5) compared to all deregulated hits in the respective brain region is shown in panel **D.** and **E.** Figure D shows that 0 (0%), 1 (25%), 10 (10%) and 1 (8.3%) unmodified proteins from all unmodified deregulated proteins of hippocampus, neocortex, olfactory bulb and brainstem, respectively, are grouped into this class. Figure E shows that 16 (41%), 16 (30.2%), 36 (28.1%) and 42 (36.2%) phosphorylated/N-linked glycosylated proteins from all phosphorylated/N-linked glycosylated deregulated proteins of hippocampus, neocortex, olfactory bulb and brainstem, respectively, are grouped into this class. The class “cytoskeleton-based synaptic plasticity” is based on the protein affiliations into sub-protein classes obtained from the PANTHER software (PANTHER protein class) as shown in Table S1–S5 (PANTHER protein classes are highlighted in yellow) involving cytoskeleton-associated processes of tubulin and actin. Principal component analysis (PCA) is shown in panel **F.** and **G.** OB, olfactory bulb; H, hippocampus; C, cortex; BS, brainstem; AD, Alzheimer's; WT, wild-type.

Noteworthy, protein class analysis revealed that a number of proteins function within synaptic cytoskeletal structures. Interestingly, more than 30% of all deregulated phospho- and glyco-proteins per brain region were involved in signalling pathways related to cytoskeleton-based synaptic plasticity (Figure 2E) compared to the much lower percentage found in the global proteomics approach to quantify unmodified peptides/proteins (neocortex: ~25%, olfactory bulb/brainstem: < 10% and hippocampus: 0 %) (Figure 2D). Five phospho-/glyco-proteins overlapped among the four brain regions consisting of the microtubuli-associated proteins Mapt, Map2, Map1b, Map1a and Sgip1 (Figure 2B). GO term analysis revealed that the majority of deregulated proteins on the global proteome and PTMome were associated with the cellular component of “cell projection part”, “neuron part” and “synapse” that regulate biological processes of “cytoskeleton organization”, “neuron projection development” and “axon development” (SI Figure S1).

### Cytoskeletal, synaptic and neuroprotective signalling pathways are affected in transgenic mice

**Figure 3:**
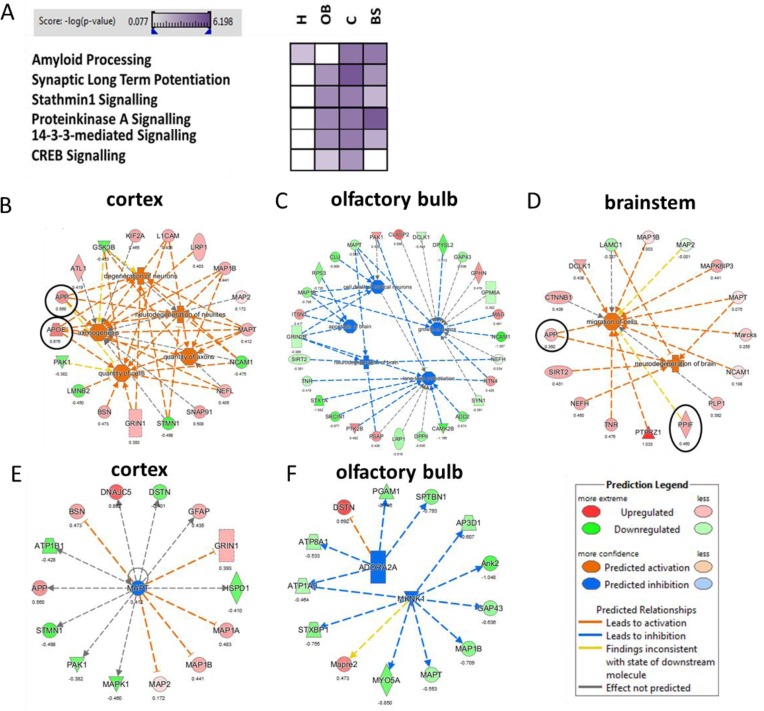
Evaluation of affected signalling pathway, biological and disease function as well as upstream regulator analysis Altered signalling pathways in the hippocampus (H), olfactory bulb (OB), neocortex C and brainstem (BS) using the Ingenuity Pathway Analysis software are shown in panel A. High color intensity represents high significance (p value) of the pathway. All colored boxes have a p value of ≤0.05; white boxes have a p value of ≥0.05 and are not significantly changed; *n* = 3 in each brain region. Significant biological / disease functions **B.** - **D.** and upstream regulator analysis **E.** - **F.** from IPA software (z-score > 2.0: predicted significant activation of node; z-score < 2.0: predicted significant inhibition of node) were grouped and visualized for neocortex, olfactory bulb and brainstem. There was no significant change in case of hippocampus. Black circle, non-modified protein; no circle, phosphorylated/N-linked glycosylated protein; protein hit in red, up-regulated expression; protein hit in green, down-regulated expression; orange node of biological function: predicted activation; blue node of biological function: predicted inhibition.Adora2a, adenosine receptor A2a; Mknk1; MAP kinase-interacting serine/threonine-protein kinase 1; Mapt; microtubuli-associated protein tau.

To obtain information about the APP/PS1-induced signalling pathways, we performed a bioinformatics analysis of all deregulated proteins (unmodified, phosphorylated and formerly sialylated N-linked glycosylated proteins) using IPA software. In spite of the relatively low number of shared deregulated proteins and modified proteins among the four brain regions, the pathways altered by APP/PS1 mutation showed a considerable overlap (Figure 3A). The signalling pathways were associated with cytoskeleton (stathmin1 signalling, amyloid processing) and synaptic neuromodulation/-transmission (14-3-3-mediated signalling, CREB signalling, protein kinase A signalling, synaptic long-term potentiation) (Figure 2A). Noteworthy, these signalling pathways were mainly affected in the olfactory bulb, neocortex and brainstem but not in the hippocampus (Figure 3A). IPA analysis of diseases and biological functions especially addresses neurodegeneration, axonogenesis, migration and LTP that are associated with synaptic structures as well as apoptosis in the neocortex, brainstem and olfactory bulb (Figure 3B-3D). No significant changes were noted in the hippocampus regarding these classes.

The analysis of upstream regulators by IPA suggested an inhibition of adenosine receptor A2a (Adora2a) signalling and MAP kinase-interacting serine/threonine-protein kinase 1 (Mknk1) in the olfactory bulb as well as a predicted inhibition of Mapt in the neocortex (Figure 3E and 3F). Significant changes of upstream regulators were not observed in hippocampus and brainstem.

### Validation of signalling pathways reveal major changes in neuronal receptors, neurotrophic factors, CREB-mediated signalling axis, stathmin1 and 14-3-3 proteins

To validate the observed deregulation of signalling pathways, biological functions and upstream regulator factors, we quantified the expression of 84 mRNA transcripts of genes that are associated with synaptic plasticity in neurons. We also performed ELISA, immunoblotting and immunohistochemistry of key proteins / metabolites involved in associated pathways to confirm their status of deregulation.

Synaptic alterations were evaluated by mRNA transcript quantification of key glutamatergic neuronal receptors and neurotrophic factors that were changed in varying extent in all brain regions. Details can be found in the Supplementary Information section (SI Table S6). The glutamate receptors in the neocortex (*Grm8*), olfactory bulb (*Grin1, Grm7*) and the glutamate receptor-interacting protein 1 (*Grip1*) were elevated whereas they were not changed in the hippocampus. The insulin-like growth factor 1 (*Igf1*) was increased in the neocortex as well in hippocampus, whereas neurotrophin 5 (*Ntf5*) and neuronal pentraxin 2 (*Nptx2*) as well as serum response factor (*Srf*) were decreased in the neocortex and hippocampus, respectively. Important modulators of synaptic transmission such as the calmodulin-dependent kinase *Camk2g* (hippocampus) and the transcription factors *Crem* (brainstem) and *Nfkb1* (neocortex) were also elevated.

**Figure 4:**
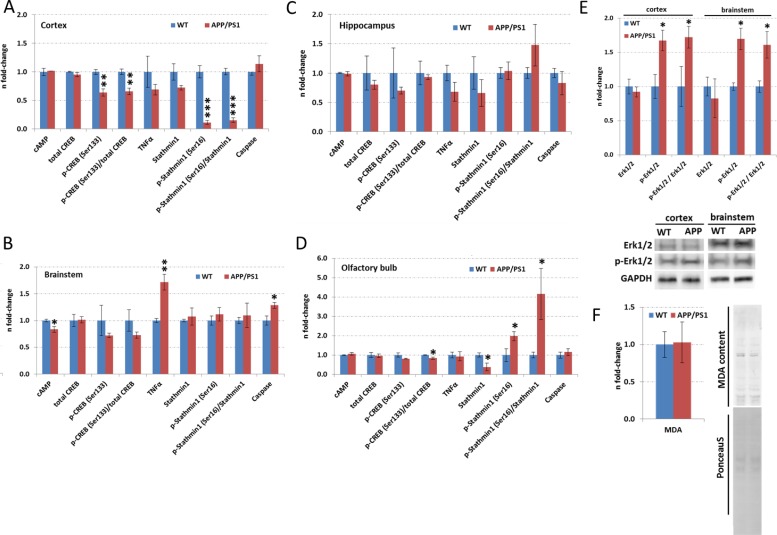
ELISA and immunoblotting of targets involved in CREB signalling, lipid peroxidation and microtubule-processing in hippocampus, neocortex, olfactory bulb and brainstem Data from ELISA of total CREB, phospho-CREB(Ser133), stathmin1, phospho-stathmin1(Ser16), TNFα, caspase3 and cAMP are depicted in panel A (neocortex), B (brainstem), C (hippocampus) and D (olfactory bulb). Panel E and F show the data from Erk1/2, p-Erk1/2 and MDA quantification using immunoblotting. The columns represent the fold-changes with standard errors of the mean (SEM); *n* = 3; *p < 0.05; **p < 0.01; ***p < 0.001 (unpaired Student's *t*-test). Normalization was performed against endogenous GAPDH for all ELISA targets, Erk1/2 and p-Erk1/2; MDA content was normalized against total lane intensity via Ponceau S staining. MDA, malondialdehyde; WT, wild-type.

**Figure 5:**
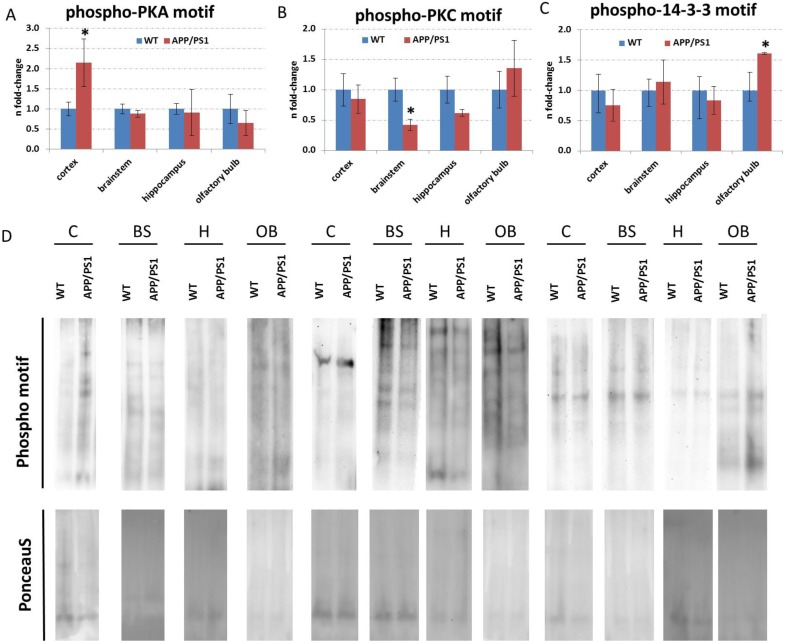
Immunoblotting of total proteins with phospho-motifs against PKA, PKC and 14-3-3 Panel A to C show the fold-changes of proteins with phospho-motifs against PKA, PKC and 14-3-3, respectively. The columns represent the fold-changes with standard errors of the mean (SEM); *n* = 3; *p < 0.05; **p < 0.01; ***p < 0.001 (unpaired Student's *t*-test). Normalization was performed against total lane intensity *via* Ponceau S staining. Panel D shows representative images of the immunoblotting following panel A-C with corresponding Ponceau S stainings. C, neocortex; BS, brainstem; H, hippocampus; OB, olfactory bulb; WT, wild-type.

As CREB signalling was indicated to be changed by the APP/PS1 mutation (Figure 3A), we quantified total CREB and p-CREB (Ser133) levels using ELISA. We noted significantly reduced expression of p-CREB (Ser133) levels compared to total CREB levels (p-CREB (Ser133) / total CREB) in the neocortex and olfactory bulb (Figure 4A and 4D). However, there was no alteration in total CREB and total *Creb* mRNA transcript expression in any brain region (Figure 4A-4D). This is consistent with data from IPA signalling pathway analysis regarding CREB signalling in the olfactory bulb and neocortex (Figure 3A) and Adora2a signalling inhibition in the olfactory bulb (Figure 3F). The associated signalling pathways of Adora2a have CREB as a common downstream transcription factor target as revealed by IPA. Next, we studied the pathway nodes and key modulators upstream of CREB in the APP/PS1 brains. We found a significant reduction in cAMP levels only in the brainstem (Figure 4B) and an increase in Erk1/2 (MAPK) phosphorylation (p-Erk1/2 / Erk1/2) in the neocortex and brainstem (Figure 4E). The activity of PKA and PKC signalling was evaluated by phospho-motif immunoblotting. We noted a global increase in phosphorylated proteins with PKA and PKC motif in the neocortex and brainstem, respectively (Figure 5A, 5B and 5D).

To validate stahmin1 signalling and 14-3-3-mediated signalling obtained from IPA analysis (Figure 3A), we performed ELISA and immunoblotting. The results suggested an increase in 14-3-3 activity in the olfactory bulb (Figure 5C and 5D) as noted by studying the global phospho-protein levels with phospho-14-3-3 motif. Interestingly, 14-3-3-mediated signalling was not changed in any brain region by quantification of *Ywhaq (14-3-3 protein theta)* but the protein expression of Ywhaz (14-3-3 protein zeta/delta) as well as Ywhae (14-3-3 protein epsilon) were downregulated in the olfactory bulb whereas their phosphorylation status was increased. We also noted a decreased phosphorylation of Ywhag (14-3-3 protein gamma) in the brainstem. Moreover, we observed a reduction in p-stathmin1 (Ser16) levels in the neocortex (Figure 4A) whereas there was both an increase in p-stathmin1 (Ser16) and a decrease in stathmin1 levels in the olfactory bulb (Figure 4D) reflecting the pathway changes of stathmin1 signalling seen in neocortex and olfactory bulb by IPA (Figure 3A). This is congruent with our proteomics data (neocortex: unchanged Stmn1 levels, decreased phosphorylation of Stmn1 based on single phospho-peptide (Ser16); olfactory bulb: reduced Stmn1 levels, increased phosphorylation of Stmn1 based on 3 phospho-peptides (Ser16, Ser25; Ser16; Ser38) (SI Table S1, S2, S5). We also observed an increase of hippocampal p-Stathmin1 (increase in a single phospho-peptide on Ser63) whereas no changes in phospho-Stathmin1 were noted in the brainstem. As IPA analysis of diseases and biological functions address neurodegeneration and apoptosis in the neocortex, brainstem and olfactory bulb (Figure 3B-3D), we quantified levels of activated Caspase-3, which was only significantly elevated in the brainstem (Figure 4B).

### Argyrophilic tangle-like structures occur in 12 month old APP/PS1 mice

**Figure 6:**
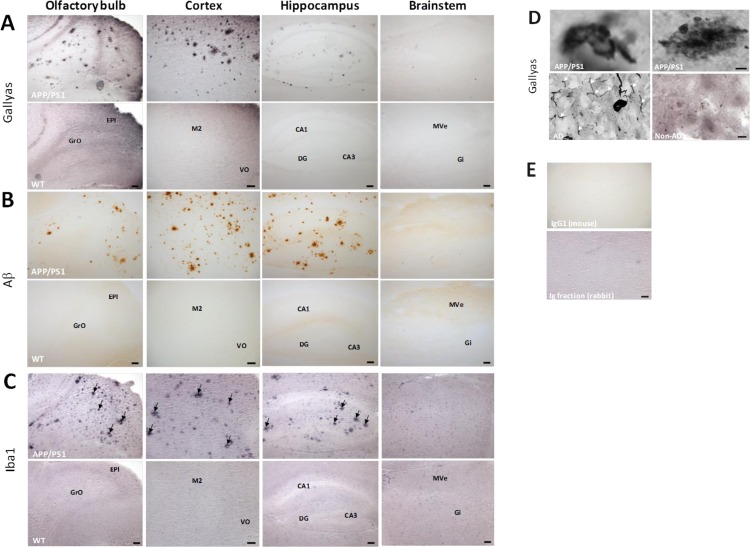
Immunostaining of argyrophilic tau protein, Aß plaques and microglia clusters and specificity of Gallyas staining using human post-mortem brain samples 20 μm-thick sagittal sections from transgenic and wild-type animals were stained for argyrophilic tau (A; Gallyas silver stain), Aβ (B; 6E10), and microglial cells (C; Anti-iba1). **A.** Gallyas-positive lesions were observed throughout the neocortex, in the olfactory bulb and hippocampus, but not the brainstem of transgenic APP/PS1 mice. The inserts show representative magnifications of cortical argyrophilic lesions, observed either isolated (left insert) or within a brown background (right insert). **B.** Aβ levels were high in the neocortex and the hippocampus of APP/PS1 mice. 6E10 immunoreactivity was observed in the olfactory bulb, but not the brainstem of transgenic animals. **C.** Arrows point to clusters of microglia in the olfactory bulb, neocortex, and the hippocampus of APP/PS1 mice. Scale bars: 100 μm (olfactory bulb, hippocampus, and brainstem); 200 μm (neocortex). GrO, granular cell layer of the olfactory bulb; EPI, external plexiform layer of the olfactory bulb; M2, secondary motor neocortex; VO, ventral orbital neocortex; CA1, field CA1 of hippocampus; DG, dentate gyrus; CA3, field CA3 of hippocampus; MVe, medial vestibular nucleus; Gi, gigantocellular reticular nucleus. Data represent from 3-4 biological replicates. **D.** To confirm the specificity of the Gallyas staining for abnormal tau in APP/PS1 mice, snap-frozen autopsy specimens from the neocortex of an AD patient and an aged-matched control were sectioned at 20 μm, and stained in parallel with mouse tissue. Argyrophilic neuropil threads (white arrows) were not observed in APP/PS1 mice. Scale bar: 10 μm.**E.** Representative images of isotype controls. All controls were diluted to the same protein concentration as the primary antibodies, and all sections were processed in parallel. Scale bar: 100 μm.

As stathmins interact with microtubules and microtubule-associated protein tau (Mapt) [14], we used a modified Gallyas method enabling us to visualise argyrophilic structures, including NFTs in AD. NFTs derive, in part, from cytoskeletal proteins with the phosphorylated tau protein being particularly abundant in these structures [15]. Argyrophilic lesions were observed in the neocortex of transgenic mice as well asolfactory bulb and hippocampus, respectively (Figure 6A). Gallyas-positive argyrophilic structures were frequently detected within a dark brown background. The argyrophilic structures present in the APP/PS1 mouse brain resembled those in post-mortem preparations from the neocortex of an AD patient (Figure 6D). However, argyrophilic neuropil threads were observed in human post-mortem brain but not in APP/PS1 mice (Figure 6D). No argyrophilic lesions were observed in the brainstem of transgenic animals or the brain of wild-type controls (Figure 6A). Data from phospho-proteomics experiments indicated an altered phosphorylation pattern of Mapt in the olfactory bulb (7 phospho-peptides), neocortex (1 phospho-peptide), brainstem (2 phospho-peptides) and hippocampus (3 phospho-peptides) (SI Table S2 to S5).

Since the APP/PS1 mouse is essentially a model for cerebral amyloidosis, we also visualized Aβ plaques by the 6E10 antibody raised against human Aβ. Variable-sized Aβ plaques were observed throughout the neocortex of transgenic mice (Figure 6B). 6E10 immuno-positive plaques were also present in the hippocampus of APP/PS1 mice (Figure 6B). Aβ plaques were further noted in the olfactory bulb, but not in the APP/PS1 brainstem or wild-type mice (Figure 6B). Proteomics data indicated an increase in Amyloid beta A4 protein (App) in neocortex, hippocampus and brainstem but not in the olfactory bulb as the fold-change was only 0.93 and is not reaching our set proteomics analysis parameters (SI Table S1). The identified App peptides correspond to both human and murine peptide sequences.

### APP/PS1 leads to changes in miRNA expression and modulation of neuroinflammatory processes

**Figure 7:**
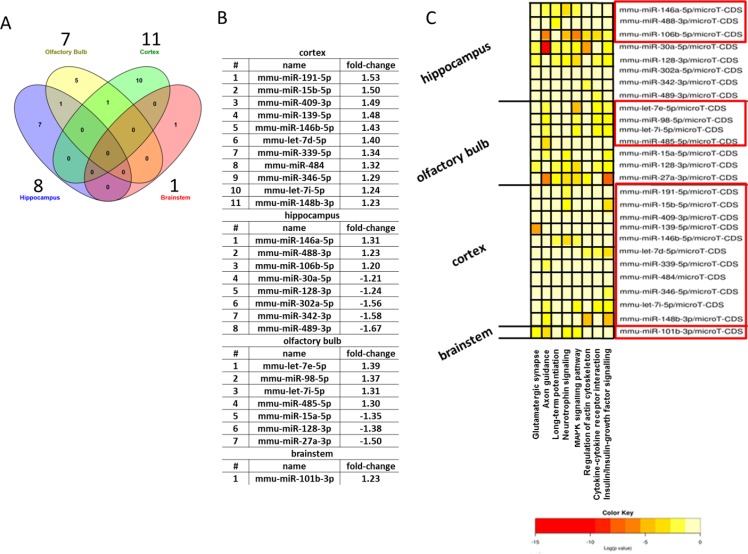
Analysis of neurological miRNAs in the brain regions Venn diagram showing the number of all and shared deregulated miRNAs in the hippocampus, olfactory bulb, neocortex and brainstem in 12 month old APP/PS1 male mice **A.**; *n* = 3. The number above each brain region shows the total number of significantly changed miRNAs per brain region. Panel B shows each significant deregulated miRNA with its fold-change per brain region. Panel C shows the significantly deregulated miRNAs (red box: up-regulated; no box: down-regulated) per brain region associated to signalling pathways in a heat map presentation.

Epigenetic regulation via miRNAs is rapidly recognized as central regulators of gene and protein expression in the postnatal mammalian brain. Thus, we studied the profile of 84 miRNAs associated with neurological development and neurological cognitive diseases such as autistic disorders, schizophrenia, AD and Huntington's disease. Details can be found in the Supplementary Information section (SI Table S7). We found in total 8, 7, 11 and 1 miRNAs significantly regulated in the hippocampus, olfactory bulb, neocortex and brainstem, respectively (Figure 7A and 7B). Only 2 deregulated miRNAs overlapped between the brain regions consisting of the miR-128-3p (down-regulated in hippocampus and olfactory bulb) and let-7i-5p (up-regulated in neocortex and olfactory bulb) (Figure 7A and 7B).

Figure 7C shows that a number of miRNAs are associated to signalling pathways involved in synaptic plasticity (glutamatergic synapse, axon guidance, long-term potentiation, MAPK signalling pathway, regulation of actin cytoskeleton) as well as inflammatory modulation (insulin/insulin-like growth factor signalling, neurotrophin signalling, cytokine-cytokine receptor interaction). The involvement of inflammatory processes in hippocampus, olfactory bulb and neocortex but not brainstem (Figure 7C) is in good agreement with the observation of clusters of microglia stained with anti-iba1 antibody in the hippocampus, olfactory bulb and neocortex, whereas no microglial clusters were noted in the brainstem of transgenic mice (Figure 6C). Anti-iba1 stained visibly more intensely in APP/PS1 brain sections compared with control tissue (Figure 6C). Contrastingly, we noted only an increased TNFα level in the brainstem (Figure 4B) that was not associated to persistent oxidative stress (quantification of lipid peroxidation by total MDA-modified protein content) (Figure 4F).

### Integrative network analysis of altered proteome, PTMome as well as pathway-focused mRNAs and neurological miRNAs

**Figure 8:**
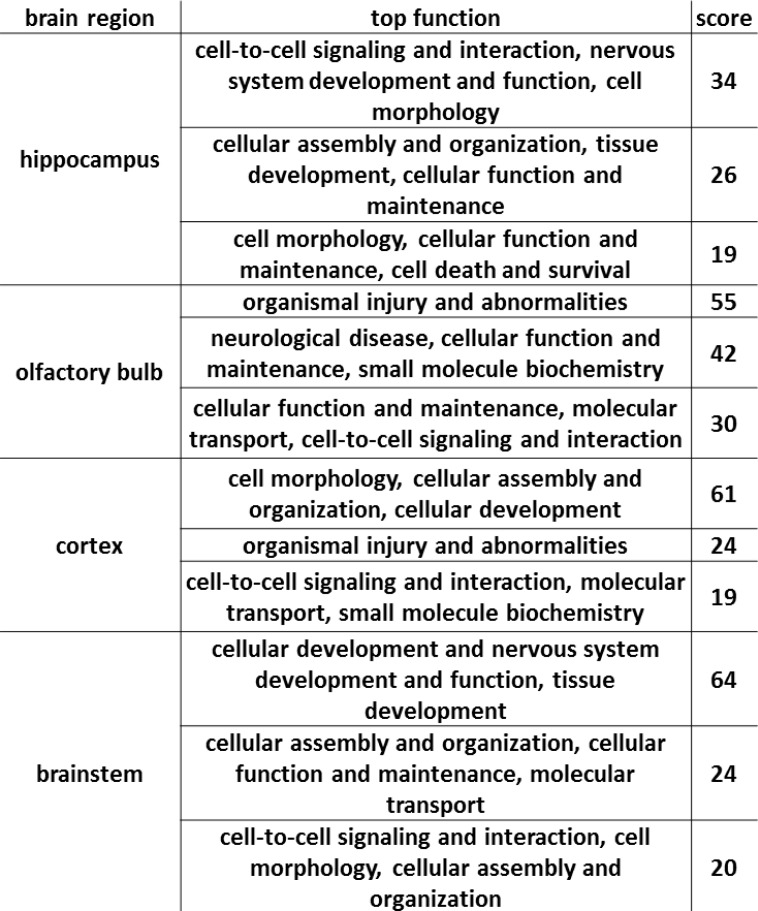
Network integration of miRNA, mRNA and proteome data The figure shows the top three biological/molecular functions of the networks in the hippocampus, olfactory bulb, neocortex and brainstem with the IPA scores.

The combined network analysis showed that the mRNA and particular miRNA data built a network with the deregulated unmodified and modified proteins (SI Figure S2 and S3). Changes in molecules associated with the extracellular space, plasma membrane, cytoplasm and nucleus were involved in all the brain regions in the APP/PS1 mouse (SI Figure S2 and S3). The top three functions of these networks were in particular associated with cellular morphology and cell-cell signalling (Figure 8) in all the four brain regions that suggest neurodegeneration and a manifestation of altered synaptic plasticity.

## DISCUSSION

### APP/PS1 mutation affects synaptic stathmin-mediated cytoskeletal dynamics

The aberrant phosphorylation of stathmin1 in one or more of its four serine residues (Ser16, Ser25, Ser38 and Ser63) reduces its microtubule instability activity [16] with Ser16 and Ser63 as the main contributors [17]. These phospho-sites were affected in our phospho-proteomics approach and are congruent with ELISA data of phospho-stathmin (Ser16). Interestingly, a reduced stathmin1 expression has been noted in the frontal and temporal neocortex of AD and Down syndrome patients [18]. Because stathmin1 functions as a key regulator of microtubule dynamics in dendrites [19], we suggest that the APP/PS1 mutations induce changes in dendritic microtubule cytoskeletal processing and dendrite arborisation, particularly in the neocortex and olfactory bulb. A dysregulated stathmin1 signalling, as indicated in the proteome analysis, cannot be ruled out in the APP/PS1 hippocampus. This is also highlighted by alterations in the phosphorylation level of certain septins in the olfactory bulb (Sept4, Sept6) and microtubule-associated proteins (Map's) in all four brain regions. Septins are critical for spine morphogenesis and dendrite development [20]. Additionally, we observed a number of cytoskeletal neurofilament proteins altered in their phosphorylation pattern (olfactory bulb: Nefh; brainstem: Nefh, Nefm; cortex: Nefl).

We also showed changes in the sialylated N-linked glycosylation and phosphorylation pattern of neural / neuronal cell adhesion molecules (Ncam1, −2 and Nrcam) in all four APP/PS1 brain regions. They regulate neurite outgrowth and synapse modulation [21]. Notably, Aß affects synapse loss in AD hippocampus by disruption of Ncam2 [22] and overexpression of wild-type PS1 or PS1 with a familial AD mutation (M146L) leads to decreased sialylation of Ncam in the neuroblastoma cell line SH-SY5Y [23]. Recently, Hondius et al. demonstrated that the human post-mortem AD hippocampus has an altered expression of a number of proteins associated with extracellular matrix components and calcium-dependent signalling proteins that are linked to early changes in cytoskeletal dynamics and synaptic plasticity over the different Braak stages compared to matched controls [24].

Generally, changes in spine shape, -size and number depend on local cytoskeletal dynamics and signalling with synaptic activity being one of the most important factors that regulate Aβ levels [6].

Importantly, the regulation of Aβ levels also occurs at epigenetic level. It has been shown that miR101 overexpression significantly reduces App and Aβ load in hippocampal neurons [25]. Interestingly, a microRNA responsive element for miR101 was identified in the 3′-untranslated region (UTR) of App [25]. We noted an increase in miR101-3p in the brainstem that may be sufficient to prevent Aβ plaques in the brain. Further, overexpression of miR106b negatively regulates the expression of endogenous App in cell lines targeting APP directly [26]. Our data indicate an upregulation of miR106b only in the hippocampus acting as a presumptive adaptive response to balance the increased App protein levels in our transgenic mouse model. However, some studies have also related miR106b to apoptosis in AD [27], but we exclude its role in apoptosis based on unchanged activated caspase-3 data in our study.

### APP/PS1 mutations induce neurofibrillary tangle-like structures

We demonstrated that NFT-like structures are apparent in 12 months old APP/PS1 mice in the neocortex, hippocampus and olfactory bulb but not in the brainstem. The argyrophilic structures were associated with a deregulated phosphorylation-pattern of tau protein (Mapt). Despite the scarcity of information regarding tau-related pathology in APPswe/PS1ΔE9 mice, the aberrant phosphorylation of tau in this mouse model has been reported previously. For instance, phospho-tau has been observed in 6 and 9 months old APP/PS1 mice using immunohistochemistry with phospho-specific antibodies, targeting tau residues S199, S202, S262 and T191 [28]. In addition, assessment of tau hyperphosphorylation by immunoblotting in 3-7 months old APP/PS1 mice indicates that tau PMTs appear shortly after plaque deposition in this mouse model, and increase robustly with age [29] [30]. As age is an important contributing factor to neurodegeneration in general and AD in particular, we speculate that the early phosphorylation of Mapt in APPswe/PS1ΔE9 mice may underline the appearance of the Gallyas-positive argyrophilic structures observed here at 12 months of age. Thus, our study does not only confirm previous reports on the phosphorylation of ‘wild-type’ tau in the APPswe/PS1ΔE9 mouse model, but also extends these observations to the proteomics level in four different brain regions, inviting further consideration of the mechanisms linking amyloidosis and tau aggregation in animal models of AD, as previously discussed [31].

The present data implicate several microtubule-regulating proteins in generating the argyrophilic structures in APPswe/PS1ΔE9 mice. Stathmin, for instance, was deregulated in APP/PS1 mice in several brain regions, most notably the neocortex and olfactory bulb, Of note, co-expression of stathmin with tau protein in COS-7 cells leads to microtubuli disruption and subsequent tau protein phosphorylation [32]. In addition, proteins such as 14-3-3, which play an important role in the abnormal fibrillization of tau in the AD brain [33], interacting with protein kinases and tau to facilitate its phosphorylation [34], were shown here to be deregulated in a region-specific manner. This suggests that an aberrant expression level and/or activity of 14-3-3 proteins in the olfactory bulb of both AD patients and APP/PS1 mice may link to NFT-like structures. In agreement with this study, Zelaya et al. noted a decrease in 14-3-3 proteins in the olfactory bulb of human post-mortem AD cases using proteomics [35]. They found an impressing number of deregulated proteins (231) that are involved in dendritic morphogenesis, neuronal injury and axonic distribution. The targets of this human study correlate well with our murine results.

Although olfactory dysfunctions are evident in neurodegenerative diseases such as AD (90% in all cases) [36], few studies have examined this area using high throughput molecular approaches such as proteomics; data on the phosphorylation and N-linked sialylated glycosylation of proteins are even more underrepresented. Thus, our integrative approach and molecular data particularly in the olfactory bulb will support the identification of potential AD biomarkers.

### APP/PS1 mutation leads to a brain-region specific response in CREB-mediated synaptic signalling and neuroinflammation

**Figure 9:**
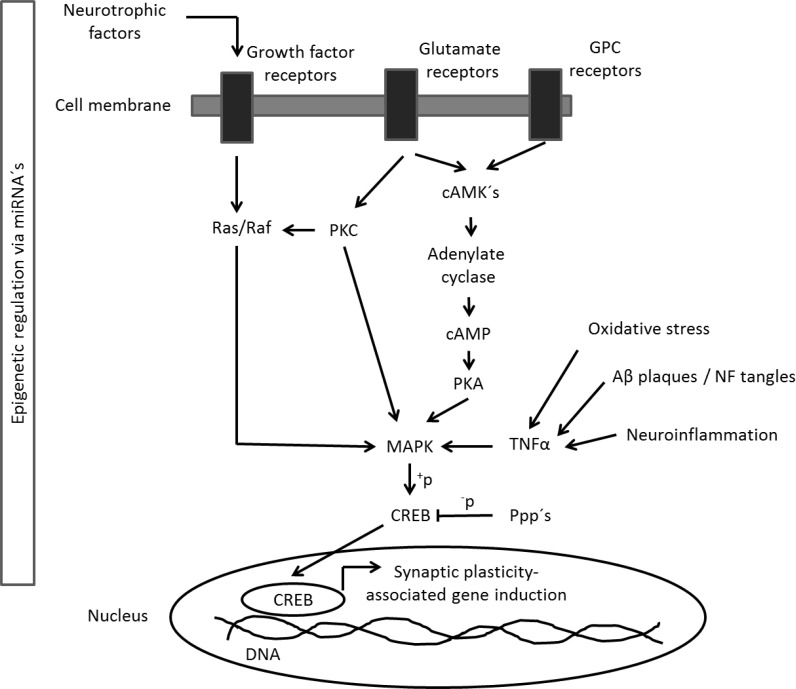
CREB-mediated signalling pathway The figure shows the simplified CREB signalling pathway in neurons. Activated neuronal receptors (Growth factor-, Glutamate- and GPC receptors) intracellularly signal to kinase proteins (Ras/Raf, PKC, cAMK's) phosphorylating MAPK proteins. This leads to phosphorylation of the transcription factor CREB shuttling to the nucleus and leads to transcription of genes that regulate synaptic plasticity. The signalling pathway can be regulated by miRNAs, Ppp's and inflammatory cytokines such as TNFα triggered by oxidative stress, Aβ plaques / NF tangles or neuroinflammation. Ppp's, protein phosphatases; GPC, G-protein coupled; NF, neurofilament.

The activation of CREB-mediated signalling pathways is necessary for the formation of synaptic long-term memory [37, 38] controlling microtubule dynamics [39]. In detail, the protein kinases cAMK's, PKA, PKC and MAPK, as well as several protein phosphatases (Ppp's) regulate CREB activity by phosphorylation [40] (Figure 9). We noted a reduced phosphorylation of CREB at the main regulatory phospho-site (Ser133) only in the neocortex and olfactory bulb. An impaired CREB phosphorylation has been described in murine AD model [41] and human AD patients [42]. Our proteomic data indicated a deregulation on the phosphorylation level of the upstream kinases of CREB in the neocortex (downregulation of Camk2b, Prkcg, Mapk1; upregulation of Ppp3cb) and olfactory bulb (downregulation of Camk2b, Prkcb, Prkce and Ppp1cb). We demonstrated an increase in MAPK signalling and a suggested increased PKA- but unchanged PKC-signalling activity in the neocortex but not in the olfactory bulb, although we found a reduced phosphorylation status of Prkc's using proteomics. The olfactory bulb-related alterations in these pathway axes culminating in CREB may be explained by expression changes of neuronal receptors on mRNA, protein and PTM level for growth factors (olfactory bulb: decreased Grb2, glyco-Lrp1; increased p-Gpr37l1; neocortex: increased glyco-Lrp1) and glutamate (olfactory bulb: decreased p-Grin1, *Grin1, Grm7;* neocortex: p-Grm2, glyco-Grin1, *Grm8*).

We conclude from this data, that the APP/PS1 mutation may lead to a reduced phosphorylation of cortical CREB that seems not to be balanced by induction of upstream regulation. The molecular mechanism of p-CREB reduction in the olfactory bulb may include changes in the neuronal receptor profile, Camk2b expression and protein phosphatase processing. The hippocampus seems to be unaffected regarding CREB signalling congruent with signalling pathway analysis of proteomics data. Interestingly, p-CREB levels seem to be unchanged in the brainstem, although we found a reduction in the cAMP metabolite. Notably, cAMP controls APP metabolism and Aß production as previously shown in neuronal cells [43]. Proteomics showed a number of phosphorylation changes in protein phosphatases in the brainstem (upregulation of Ppp3cb and Ppp3ca), protein kinases (upregulation of Prkcb and Prkar2b; downregulation of Prkce) and downregulation of Mapk3. Using immunoblotting, we found that PKC but not PKA signalling is inhibited in the brainstem by APP/PS1 mutation. Moreover, we noted an upregulation of MAPK signalling (p-Erk1/2). It is known that the MAPK cascade involves both PKA and PKC signalling to CREB phosphorylation in the brain [44] (Figure 9). Thus, these results are congruent with IPA signalling pathway data.

There are a number of evidences that an elevated MAPK activation is involved in early stages of AD [45]. Importantly, the activation of MAPK signalling occurs via cellular stress arising from inflammatory cytokines such as TNFα [46] or Aβ plaques [47]. We showed that elevated phosphorylation of Erk1/2 in the neocortex and brainstem is associated with Aβ plaques and increased TNFα expression, respectively, as well as increased apoptosis-inducing caspase3 levels in the brainstem. In contrast, we demonstrated that neuroinflammation (microglial clusters positive for iba1) exists in all analyzed brain regions except brainstem. As we used total brain region homogenates for TNFα and caspase3 quantification, we cannot target these factors to a specific cell type. However, microglia seem to be their main source in other neurological conditions in mice [48] and in 12 month old APP/PS1 mice [12]. Interestingly, in the APP/PS1 mouse, TNFα was shown mainly to be expressed in those microglia, that had not taken up Aβ [12]. We also found increased neurotrophic *Igf1* levels in the transgenic neocortex and hippocampus. Igf1 plays a trophic role in tissue injury with microglia being an important source of it [49]. Interestingly, a link between inflammation, neuronal dysfunction and defective insulin signalling has been demonstrated in AD [50] whereas Igf1 has also a major role in dendritic outgrowth [51]. Recent work to elucidate neuroinflammatory signals in APP/PS1 mice and AD in human beings emphasizes that the inflammatory changes in APP/PS1 mice coincided with Aβ deposition [52] [12]. Moreover, it was shown that major changes in the mRNA expression of immune response mediators, including cytokines, in the brain of wildtype mice occurred with ageing, which in humans is coincidental with the first alterations of sporadic AD-related pathology [52].

Regarding miRNA, miR146a regulates neuroinflammation [53]. A number of evidences point to its increased expression in APP/PS1 transgenic mice, such as in the neocortex of 6 month-old mice [54] and in the hippocampus and neocortex of AD patients [55, 56]. The increased expression of miR146 in the hippocampus (miR146a-5p) and neocortex (miR146b-5p) correlates with the presence of clusters of iba1-positive microglia in these brain regions. It is unclear whether miR-146 is an inhibitor of immune response and might be protective in neurodegenerative diseases [57].

Overall, we conclude that the olfactory bulb, neocortex, brainstem and hippocampus have a distinctively different molecular signature in male APP_swe_/PS1ΔE9 mice, which might explain their differential sensitivity to AD-related pathology. It remains to be seen whether these changes correspond to post-mortem brains of human AD patients.

## MATERIALS AND METHODS

### Ethics statement and tissue collection

Mouse brain tissue. Experiments were performed according to protocol number J.nr. 2011/561-1950 approved by the Danish Animal Ethics Inspectorate. Male APP/PS1 (B6.Cg-Tg(APPswe, PSEN1dE9)85Dbo/Mmjax) line 85 mice [58] and littermate wild-type mice were bred in the Biomedical Laboratory, University of Southern Denmark under a 12:12h light:dark cycle (lights on 6:30 am). Food and water were available ad libitum. Genotyping was performed in-house. Mice were sacrificed by cervical dislocation at the age of 12 months. Brains were excised and the left hemisphere was dissected for olfactory bulb, hippocampus, neocortex and the brainstem. The brain regions were immediately heat stabilized using the Stabilizor^TM^ T1 (Denator). The right hemisphere was snap-frozen in isopentane on dry-ice for staining experiments (−30°C). All brain samples were stored at −80°C until further processing. In total, 6 mice were used (3 mice per group).

Human brain tissue. As control for staining experiments on the mouse brain tissue, we used samples of frontal neocortex from AD patients (BB08-002) and a control case (BB07-015). The human brains were obtained from The Maritime Brain Tissue Bank, Department of Medical Neuroscience, Faculty of Medicine, Dalhousie University, Sir Charles Tupper Building, 5850 College Street, Halifax Nova Scotia B3H 1X5 following approval from the Danish Biomedical Research Ethical committee for the Region of Southern Denmark (Project Id. S-20070047) and the Nova Scotia Health Authority Research Ethics Board in Halifax, Canada. Informed, written consent forms were obtained from both donors. Brains were removed ≤24 hours post-mortem and frozen at −80°C.

### Isolation of total protein and RNA

The PARIS^TM^ kit (Ambion) was used to isolate proteins and total RNA from the same sample. Tissue samples were homogenised in manufacturer's Cell Disruption Buffer including protease and phosphatase inhibitors (cOmplete Protease Inhibitor and PhosSTOP, Roche Diagnostics) on ice using a manual plastic mortar. An aliquot of the homogenate was saved for proteomics experiments (mass spectrometry, ELISA and immunoblotting) and stored at −20°C. The other part of the homogenate was processed for RNA isolation using manufacturer's instructions and purified by RNeasy MinElute Cleanup Kit (Qiagen); RNA was eluted in nuclease-free water. The optical density (OD) ratio of 260/280 was measured using a Nanodrop spectrophotometer (PeqLab Biotechnology); it ranged between 1.9 and 2.0. RNA samples were stored at −20°C.

### Mass spectrometry-based proteomics

The complete sample processing workflow is depicted in Figure 1A. Figure 1B shows the number of identifications and quantifications of the proteomics experiments.

### Reduction, alkylation and enzymatic digestion

150 μg total proteins were denatured and reduced in 6 M urea, 2 M thiourea, 10 mM DTT, 20 mM TEAB, pH 7.5 at room temperature (RT). After vortexing and sonication, proteins were alkylated in 20 mM iodoacetamide (IAA) for 20 minutes in the dark. A total of 2 μl of endoproteinase Lys-C (6 μg/μl, Wako) was added to the protein sample and the solution was incubated for 2 hours at RT. The sample was diluted 10 times with 20 mM TEAB, pH 7.5 and digested with trypsin (1:50 (w/w) trypsin:protein) overnight at 37°C. The enzymatic digestion was stopped with 5% formic acid (FA) and the peptide sample was cleared by centrifugation (14000 × g, 15 minutes). Protein and peptide quantification was performed by fluorometric quantification (Qubit^TM^ – Life technologies).

### ITRAQ labelling

A total of 75 μg tryptic peptides per brain region and condition were dried and desalted with R2/R3 columns (as described under Sample desalting with R2/R3 micro-column) before iTRAQ-8plex labelling (AB Sciex). Labelling was performed as follows: iTRAQ-113 for hippocampus wild-type, iTRAQ-114 for hippocampus AD mouse, iTRAQ-115 for neocortex wild-type, iTRAQ-116 for neocortex AD mouse, iTRAQ-117 for olfactory bulb wild-type, iTRAQ-118 for olfactory bulb AD mouse, iTRAQ-119 for brainstem wild-type and iTRAQ-121 for brainstem AD mouse. Three biological replicates were used. The labelling was performed according to manufacturer's instruction. The labelled peptides of each wild-type and mutated hippocampus, neocortex, olfactory bulb and brainstem were mixed 1:1:1:1:1:1:1:1, dried down and stored for further enrichment and analysis.

### Enrichment of phospho-peptides and formerly sialylated N-linked glycopeptides (deglycosylated)

Multi- and monophosphorylated peptides as well as sialylated N-linked glycopeptides were separated from unmodified peptides using a TiO_2_-SIMAC-TiO_2_ (TiSH) workflow: Modified peptides bind to TiO_2_ beads because the phospho and sialic groups are acid and hence retained in the column, whereas unmodified peptides flow-through; following SIMAC (sequential elution from IMAC beads) [59], multiphosphorylated peptides are enriched and eluted separately from the monophosphorylated and deglycopeptides that are, in turn, separated in a second TiO_2_ step [60–63] (Figure 1A). The eluted modified peptides from the first TiO_2_ step were deglycosylated to remove N-linked glycans [61]. Hydrophilic interaction chromatography (HILIC) was used as sample fractionation prior to nano liquid chromatography-tandem mass spectrometry (LC-MS/MS).

Briefly, the combined labelled peptides were dissolved in TiO_2_ loading buffer (80% acetonitrile (ACN), 5% trifluoroacetic acid (TFA) and 1 M glycolic acid) and incubated with 3.6 mg of TiO_2_ (5020 titansphere TiO_2_, 5μm; a kind gift from GL Sciences, Japan) beads for 30 minutes at RT. The beads were sequentially washed with TiO_2_ loading buffer, 80% ACN/1% TFA and 10% ACN/0.1% TFA. Modified peptides were eluted with 1.5% ammonium hydroxide solution, pH 11.3, and dried. The unbound TiO_2_ fraction and the combined washing fractions contain unmodified peptides. The dried modified peptides were deglycosylated in 20 mM TEAB, pH 8.0 with N-glycosidase F (P0705L, Biolabs) and Sialidase A (GK80046, Prozyme) at 37°C overnight. The sample was dried and resuspended in SIMAC loading buffer (50 % ACN, 0.1% TFA) and incubated with PhosSelect IMAC beads (P9740, Sigma) pre-equilibrated in SIMAC loading buffer. After 30 minutes under gentle shaking at RT, beads were washed in SIMAC loading buffer and monophosphorylated / deglycopeptides were eluted with 20% ACN, 1% TFA and combined with the washing fraction (SIMAC FT) and dried; multiphosphorylated peptides were eluted with 1.5% ammonium hydroxide solution, pH 11.3, and dried down. The second TiO_2_ step was performed with the SIMAC FT and 1% TFA elution. All the eluates were dried and desalted on micro-columns before capillary HILIC fractionation.

### Sample desalting with R2/R3 micro-column

The samples were desalted before HILIC fractionation using home-made P200-tip-based columns packed with equal ratios of Poros R2 (Oligo R2 Reversed Phase Resin 1-1112-46, Applied Biosystems) and Poros R3 (OligoR3 Reversed Phase Resin 1-1339-03, Applied Biosystems) reversed-phase resin material. The end of the tip was blocked with C_8_ material (Model 2314, 3m EmporeTM C8). The column was prepared by short centrifugation (1000xg) of the R3 reversed-phase resin (100% ACN). The column was equilibrated with 0.1% TFA and centrifuged again. The acidified samples were loaded onto the columns and washed / centrifuged three times with 0.1% TFA. Peptides were eluted with 60% ACN, 0.1% TFA and dried.

### HILIC fractionation

The fractions containing mono-phosphorylated, deglycosylated and unmodified peptides were fractionated prior to nano LC-MS/MS analysis using HILIC as described previously [63, 64]. Peptides were dissolved in 90% ACN, 0.1% TFA (solvent B) and loaded onto a 450 μm OD × 320 μm ID × 17 cm micro-capillary column packed with TSK Amide-80 (3 μm; Tosoh Bioscience) using an Agilent 1200 Series HPLC (Agilent). The peptides were separated using a gradient from 100–60% solvent B (A = 0.1% TFA) in 30 min at a flow-rate of 6 μl/min. Fractions were collected every 1 min based on the UV chromatogram. Subsequently, the peptide fractions were dried by vacuum centrifugation.

### Reversed-phase nanoLC-ESI-MS/MS

The peptides (resuspended in 0.1% FA) were automatically injected and loaded on a ReproSil-Pur C18 AQ (Dr. Maisch, Ammerbuch-Entringen, Germany) in-house packed trap column (2 cm × 100 μm inner diameter; 5 μm). The peptides were separated at 250 nl/min on an analytical ReproSil-Pur C18 AQ (Dr. Maisch, Ammerbuch-Entringen, Germany) packed in-house column (17 cm × 75 μm; 3 μm) by reversed phase chromatography which was operated on an EASY-nanoLC system (Thermo Fisher Scientific, Odense, Denmark). Mobile phase was 95% ACN/0.1% FA (B) and water/0.1% FA (A). Depending on the samples, the gradient was from 1% to 30% solvent B in 80 (mono-phosphoralyated-, deglycosylated- and unmodified peptides) or 110 min (multiphosphorylated peptides), 30 - 50% B in 10 min, 50 - 100% B in 5 min and 8 min at 100% B. The nano-LC was online connected to a Q Exactive HF Hybrid Quadrupole-Orbitrap mass spectrometer (Thermo Fisher Scientific) operating in positive ion mode and using data-dependent acquisition. The Orbitrap acquired the full MS scan with an automatic gain control (AGC) target value of 3×10^6^ ions and a maximum fill time of 100 ms. Each MS scan was acquired at high-resolution (120,000 full-width half maximum (FWHM) at m/z 200 in the Orbitrap with a mass range of 400-1400 Da. The 12 most abundant peptide ions were selected from the MS for higher energy collision-induced dissociation (HCD) fragmentation (collision energy: 34 V) if they were at least doubly charged. Fragmentation was performed at high resolution (60,000 FWHM) for a target of 1×10^5^ and a maximum injection time of 60 ms using an isolation window of 1.2 m/z and a dynamic exclusion of 20 s.

### Data analysis

Raw data were searched against the Swissprot database and Uniprot mouse reference database via Mascot (v2.3.02, Matrix Science) and Sequest HT search engines, respectively, using Proteome Discoverer (v1.4.1.14, Thermo Fisher Scientific). A precursor mass tolerance of 10 ppm and a product ion mass tolerance of 0.02 Da were applied allowing not more than one missed cleavage for trypsin. Fixed modifications included carbamidomethylation of Cys and iTRAQ8-plex labeling for Lys and N-terminal. Variable modifications contained phosphorylation on Ser/Thr/Tyr and deamidation of Asn. The iTRAQ datasets were quantified using the centroid peak intensity with the “reporter ions quantfier” node. To ensure a high-confident identification of peptides, we used the Mascot percolator algorithm (q value filter set to 0.01), Mascot and Sequest HT peptide rank 1 and a cut-off value of Mascot score ≥ 22 as well as Sequest HT ΔCn of 0.1. Moreover, a cut-off value of Xcorr score for charge states of +1, +2, +3 and +4 higher than 1.5, 2, 2.25 and 2.5, respectively, were considered for further analysis. Subsequently, these peptides were filtered against a Decoy database resulting into a false discovery rate (FDR) to 0.01 (FDR < 0.01). Three biological replicates were considered for the statistical analysis. Quantification was performed on the log2-values of the measured peptide intensities and the data were normalized based on the median. Modified peptides were merged with the R Rollup function (http://omics.pnl.gov/software/danter) allowing for one-hit-wonders and using the mean of the normalized intensities for each peptide. Quantification of proteins was obtained by merging the unmodified peptides with the R Rollup function considering at least 2 unique peptides not allowing for one-hit-wonders and using the mean of the intensities. Subsequently, the mean over the experimental conditions for each peptide in each replicate was subtracted in order to merge the data from different replicates. Proteins with proteoforms were deleted during data analysis. Principal component analyses (PCA) were performed to assess sample quality when comparing the different experimental conditions (individual brain region between wild-type and APP/PS1 mice). Proteins, phosphopeptides (localized by phosphoRS node within the Proteome Discoverer software)and formerly sialylated N-linked glycopeptides with a consensus motif for N-linked glycosylation (NXS/T/C; where X # P) were considered to be significantly deregulated if they fulfilled the following criteria: (1) identification by at least two unique peptides (proteins) or at least one unique peptide (post-translationally modified peptides) in n-1 replicates (n: number of biological replicates), (2) quantification with a standard deviation (SD) of ≤ 30% between the replicates and a ratio of ≥ 1.30 or ≤ 0.77. The threshold for the ratio was calculated on the average experimental technical variance of the multiple analyses of brain technical replicates in mass spectrometry analysis [65]. Phosphorylated and deglycosylated peptides were normalized based on the protein expression in each of the replicates including the proteins with only one unique peptide to ensure that deregulation occurred on PTM level and not on protein level.

The mass spectrometry proteomics data have been deposited to the ProteomeXchange Consortium [66] via the PRIDE partner repository with the dataset identifier PXD003312 (username: reviewer10739@ebi.ac.uk; password: Fztk4CaX)

### Quantification of mRNA and miRNA via quantitative PCR

A total of 100 ng of RNA isolates were used to quantify miRNA expression (miScript miRNA PCR Array “Neurological Development & Disease” [MIMM-107Z – Qiagen]) and gene expression (RT^2^ Profiler PCR Array “Synaptic Plasticity” [PAMM-126Z – Qiagen]) levels according to the manufacturer's protocol on a StepOnePlus device (Applied Biosystems). Expression levels of miRNAs and mRNAs were calculated based on the 2^−ΔΔCt^ method with normalisation against the median of all target miRNAs / mRNAs, respectively. Changes were considered significant if they reached a p-value of ≤ 0.05 (unpaired Student's t-test, two-sided, n=3) and had a fold-change of ≥ 1.2 or ≤ −1.2. The threshold of ±1.2 is based on the previous average experimental technical variance of 8.4% of a set of 14 overlapping targets gained in the neocortex and hippocampus [65]. Thus, a threshold of ±1.2 enables confident target identification [67].

### Bioinformatics analysis of protein classes, phospho-sites and affected signalling pathways

Deregulated proteins were categorised into protein classes using PANTHER (Protein Analysis Through Evolutionary Relationships) classification system software (http://www.pantherdb.org) and the general annotation from UniProt (http://uniprot.org). Gene ontology (GO) analysis of biological processes and cellular components was performed with STRING software (http://string-db.org). The analyses of affected signalling pathways from all deregulated proteins and posttranslational modified (PTM) proteins were performed with the INGENUITY Pathway Analysis (http://www.ingenuity.com) software tool that comprises curated information from databases of experimental and predictive origin, enabling discovery of highly represented functions and pathways. The mean of the ratios of all deregulated PTM-peptides per protein was used for signalling pathway analysis. Network analysis was performed by uploading the deregulated unmodified proteins, phospho-proteins and glyco-proteins as well as miRNA and mRNA expression data. We used only the database information of experimental and predictive origin regarding central nervous system to be confident about the potential affected signalling pathways. The IPA comparison analysis takes into account the signalling pathway rank according to the calculated p-value and reports it hierarchically. The software generates significance values (p-values) between each biological or molecular event and the imported proteins based on the Fisher's exact test (p ≤ 0.05).

MiRNA data were analysed with DIANA miRPath v.2.0 software [68] and the signalling pathways were visualised in a heatmap presentation by applying a p-value threshold of ≤ 0.05 (Fisher's meta-analysis) and FDR correction (Benjamini & Hochberg).

Phospho-sites related to Alzheimer's were manually compared with known information from PhosphoSitePlus database (http://www.phosphosite.org).

### Enzyme-linked immunosorbent assay (ELISA)

A total of 5 μg of protein lysates were used to quantify total CREB (KHO0231, Invitrogen), p-CREB (Ser133) (KHO0241, Invitrogen), Stathmin1 (E03S0217, Bluegene), Caspase3 (SEA626Mu, Cloud-Clone Corp.), cAMP (KGE012B, R&D Systems) and TNFα (BMS607HS, eBioscience) using enzyme-linked immunosorbent assay (ELISA) after manufacturer's instructions. P-Stathmin1 (Ser16) was quantified by using the Stathmin1 ELISA (E03S0217, Bluegene) and primary antibody against p-Stathmin1 (Ser16) (ab47328, Abcam) and secondary detector antibody (anti-rabbit IgG, HRP-linked) (#7074, Cell Signalling). The plates were measured on a FLUOstar Omega (BMG Labtech) at the recommended wavelength of the manufacturer. All assays were normalised against GAPDH (ab176642, Abcam) as it was not changed in the proteomics and transcriptomics data. Three biological replicates from hippocampus, neocortex, olfactory bulb and brainstem were analyzed in duplicates. The mean of each technical triplicate was normalised against the mean of the representative GAPDH technical replicates. Statistical analysis between APP/PS1 and age-matched wild-type mice was performed via unpaired Student's t-test and data are presented as fold-changes with the standard error of the mean (SEM).

### Light microscopy

Fresh-frozen, 20 μm-thick sections were used in all staining experiments (mouse brain and human brain tissue). Adjacent serial sections were collected at 300 μm intervals in a Leica CM3050S cryostat (Nussloch, Germany), at −17°C. The sections were mounted onto Superfrost^TM^ Plus microscope slides, stored at −80°C, and processed within 1 month of collection.

For the Gallyas silver stain, sections were initially thaw-fixed in 4% Neutral Buffered Formalin (NBF) at 4°C for 24 h. The sections were subsequently washed in distilled water (5 × 2 min) and defatted for 1 h in a solution of chloroform/99% ethanol (1:1). Following hydration through a series of graded ethanol into distilled water (2 × 1 min: 99%, 96%, 70%), sections were stained for neurofibrillary tangles according to the Kuninaka et al. 2015 modification [69] of the Gallyas method [70]. Briefly, slides were immersed into an aqueous solution of 0.25% potassium permanganate (20 min), washed in distilled water (1min), and incubated in 1% oxalic acid (2 min). After washing in distilled water (2 × 5 min), sections were transferred into an alkaline silver iodide solution (1 min), washed with 0.5% acetic acid (2 × 5 min), and developed until the appearance of a brownish shade (~10 min). The stained sections were then washed in 0.5% acetic acid (3 min), toned with 0.1% gold chloride (10 min), and fixed with 1% sodium thiosulfate (5 min).

Aβ immunohistochemistry was performed using the biotinylated 6E10 mouse anti-human antibody (SIG-39340; NordicBiosite, Norway), as detailed previously [12]. NBF post-fixed sections were treated with 70% FA (30 min), permeabilised in 50 mM Tris-buffered saline (TBS) containing 1% Triton X-100 (TBSTx; 3 × 15 min), and blocked in TBSTx containing 10% fetal bovine serum (FBS, 30 min). The primary antibody was diluted in TBS+10% FBS buffer and applied overnight at 4°C (1:500 dilution of stock). The sections were subsequently brought to RT, washed in TBSTx (3 × 15 min), and quenched for endogenous peroxidase activity in a solution of TBS/methanol/30% water (8:1:1; 20 min). After washing in TBSTx (3 × 15 min), sections were incubated with streptavidin-conjugated horseradish peroxidase for 3 h (SA-HRP, GE Healthcare, UK). Following 3 × 10 min washes in TBS, peroxidase activity was visualised with 0.05% 3,3′diaminobenzidine (DAB) in TBS buffer, containing 0.01% H_2_O_2_.

Microglial cells were stained using the rabbit polyclonal anti-Iba1 antibody (WAKO Chemicals GmbH, Germany). Sections were fixed in 4% paraformaldehyde (PFA) [71] for 24 h at 4°C, followed by fixation in 1% PFA at RT for an additional 24 h. After 5 × 2 min washes in TBS, sections were defatted for 1 h in a solution of chloroform/99% ethanol (1:1), and hydrated through graded alcohol into TBS (2 × 1 min: 99%, 96%, 70%). Endogenous peroxidase activity was quenched using 1.5% H_2_O_2_ in TBS (30 min). Sections were subsequently washed and treated for 30 min with 0.25% sodium borohydride, to reduce unreactive aldehydes and Schiff bases. Following thorough washing in TBS, the sections were immersed in TBS+0.5% Triton X-100 (4 × 15 min), and incubated with primary antibody in the same buffer for 48 h (1:5,000 dilution of stock). After washing in TBS+0.5% Triton X-100 (4 × 15 min), the anti-rabbit EnVision+ HRP-labelled polymer was applied to the sections for 3 h (Dako, Denmark). The stain was developed using 0.05% DAB, 10 mM imidazole and 0.5% nickel ammonium hexahydrate, according to a recently published procedure [72].

After each staining protocol, sections were washed in water, dehydrated in graded alcohols, cleared in xylene, and cover-slipped with PERTEX (HistoLab, Denmark). For immunohistochemistry, omission of primary antibodies, and the mouse biotin-labelled IgG1 (ThermoFisher Scientific, Denmark) or the rabbit immunoglobulin fraction (Dako, Denmark) were used to confirm the specificity of 6E10 and anti-Iba1 for Aβ peptides and microglia cells, respectively.

### Quantification of proteins and phosphorylation-motifs by immunoblotting

Protein extracts (15 μg) were separated on 4-12% Bolt Bis-Tris gradient gels (NW04125Box, Novex) and transferred to polyvinylidene fluoride (PVDF) membranes via the TransBlot SD Semi-Dry Transfer Cell Blotter system (Biorad). The protein content on the membranes was visualised with Ponceau S. The membranes were incubated in blocking buffer (5% milk) washed and incubated overnight at 4°C with primary antibody dilutions as recommended by the manufacturer (Phospho-PKC Substrate Motif (R/KXpSX(R/K) MultiMab Rabbit Monoclonal Antibody – #6967, Cell Signalling; Phospho-(Ser) 14-3-3 Binding Motif Rabbit Antibody - #9601, Cell Signalling; Phospho-PKA Substrate (RRXS*/T*) (100G7E) Rabbit Monoclonal Antibody - #9624, Cell Signalling; rabbit polyclonal antibody against p44/42 MAPK (Erk1/2) - #9102, Cell Signalling; rabbit polyclonal antibody against phospho-p44/p42 MAPK (Erk1/2) (Thr202/Tyr204) - #9101, Cell Signalling, goat polyclonal antibody against malondialdehyde (MDA) – ab20703, Abcam and goat polyclonal antibody against GAPDH – ab9483, Abcam. The blots were washed and incubated with horseradish peroxidase-conjugated secondary antibody (anti-rabbit IgG, HRP-linked antibody - #7074, Cell Signalling or anti-goat IgG, HRP-linked antibody – ab6741, Abcam) in 5% milk for 2 hours. Subsequently, blots were washed and developed with ECL system (Luminata^TM^ Forte Western HRP Substrate, WBLUF0100, Millipore) using standard protocol from the manufacturer. Immunoblots were considered for quantification if (i) the pattern and intensity of lanes stained with PonceauS were equal and total lane intensity of the PonceauS-stained proteins was similar within the biological replicates. For phospho-motif and MDA immunoblots, the total lane intensity was selected as a representative indicator of global changes in PKA-, PKC-, 14-3-3-phospho motifs and oxidative stress, respectively. Immunoblots were quantified with TotalLab TL100 software (www.totallab.com) using software-suggested background correction. Each band was normalised against the total lane intensity obtained by Ponceau S (phospho motif immunoblots and MDA blots) or against GAPDH (phospho Erk1/2 and Erk1/2) that was not changed during our transcriptomics and proteomics analysis. Fold-changes were calculated between APP/PS1 and wild-type mice from hippocampus, neocortex, olfactory bulb and brainstem separately. In case of ERK1/2 and p-ERK1/2 quantification, we quantified one lane corresponding to ERK1 and ERK2 as well as p-ERK1 and p-ERK2, respectively. Three biological replicates were used for statistical analysis (Student's t-test, unpaired) with a significance threshold of 0.05.

## SUPPLEMENTARY MATERIALS FIGURES AND TABLES

















## Abbreviations

AD: Alzheimer's disease
NFT: neurofibrillary tangle
LTP: long-term potentiation
LTD: long-term depression
PTM: post-translational modification
ELISA: enzyme-linked immunoassay
OD: optical density
RT: room temperature
IAA: iodoacetamide
FA: formic acid
TiSH: TiO_2_-SIMAC-TiO_2_
SIMAC: sequential elution from IMAC beads
ACN: acetonitrile
TFA: trifluoroacetic acid
HILIC: hydrophilic interaction chromatography
LC-MS/MS: liquid chromatography-tandem mass spectrometry
AGC: automatic gain control
FWHM: full-width half maximum
HCD: higher energy collision-induced dissociation
FDR: false discovery rate
PCA: principal component analysis
SD: standard deviation
GO: gene ontology
IPA: Ingenuity pathway analysis
SEM: standard error of the mean
NBF: neutral buffered formalin
TBS: Tris-buffered saline
TBST: Tris-buffered saline 1% Triton X-100
FBS: fetal bovine serum
PFA: paraformaldehyde
DAB: 3,3'diaminobenzidine
PVDF: polyvinylidene fluoride
MDA: malondialdehyde
Wt: wild-type
OB: olfactory bulb
H: hippocampus
BS: brainstem
C: neocortex
DG: dentate gyrus
VO: ventral orbital neocortex
M2: secondary motor neocortex
