# Supplementary material for: An integrated proteomics approach shows synaptic plasticity changes in an APP/PS1 Alzheimer's mouse model
Source: Oncotarget. 2016 Apr 28;7(23):33627–48. doi: 10.18632/oncotarget.9092 (PMC5085108; doi:10.18632/oncotarget.9092)
Supplement: Supplementary file 1 [file oncotarget-07-33627-s001.pdf]

An integrated proteomics approach shows synaptic plasticity changes in an APP/PS1 Alzheimer´s mouse model

Supplementary Material

	GO Biological Process			GO Cellular Component		
Brain region	Name	p-value	#	Name	p-value	#
Hippocampus	neuron projection development	4.40E-09	14	cell projection part	9.33E-18	23
	cytoskeleton organization	2.77E-07	14	neuron part	1.00E-17	25
	dendrite development	1.13E-06	7	synapse	1.71E-16	20
Olfactory Bulb	nervous system development	1.58E-14	61	neuron part	9.25E-43	85
	cell projection organization	1.29E-12	38	cell projection part	9.65E-41	73
	neuron projection development	5.64E-10	28	synapse	1.22E-33	59
Cortex	neuron projection morphogenesis	1.28E-08	13	neuron part	5.54E-25	33
	axon development	1.09E-07	11	cell projection part	2.59E-22	28
	cytoskeleton organizaton	7.94E-07	14	synapse	3.56E-21	25
Brainstem	localization	1.11E-15	57	synapse	1.36E-44	53
	neuron projection development	2.44E-13	24	neuron part	1.11E-42	62
	synaptic transmission	4.18E-11	19	cell projection part	1.69E-41	55

SI Figure S1: GO term analysis

Gene ontology (GO) analysis of proteomics data is shown. The panel shows the top three hits of GO biological processes and cellular component in the hippocampus, olfactory bulb, neocortex and brainstem with the name, p-value and number of hits (#). GO term analysis was performed with the STRING software (http://string-db.org). The input data to generate this table were based on the IDs of deregulated proteins.

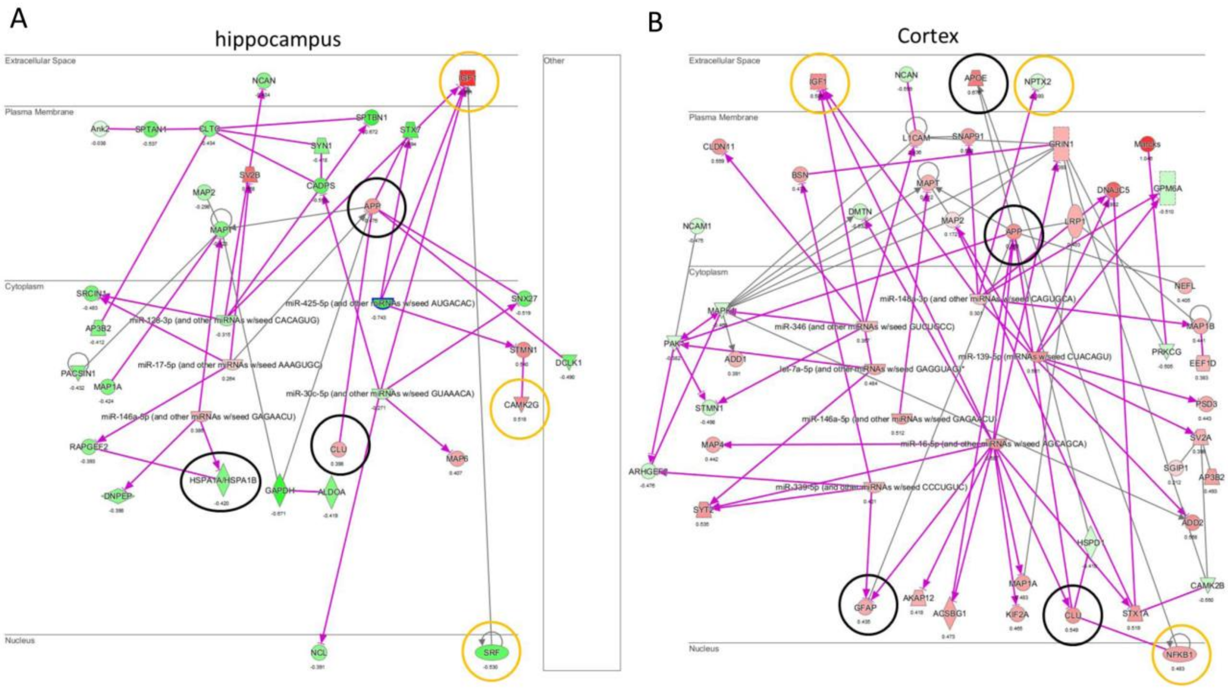

SI Figure S2: Molecular network including miRNA, mRNA, proteins and modified proteins

The figure shows the integrative networks in hippocampus (A) and neocortex (B) with the significant changed data of miRNA, mRNA and protein (non-modified, phospho- and N-linked glycosylated-proteins). Black circles: non-modified protein; orange circle: mRNA transcript; no circle: phospho-/glyco-protein or miRNA as indicated below; hit in red, up-regulated expression; hit in green, down-regulated expression. The colorcode of the arrows is irrevelant.

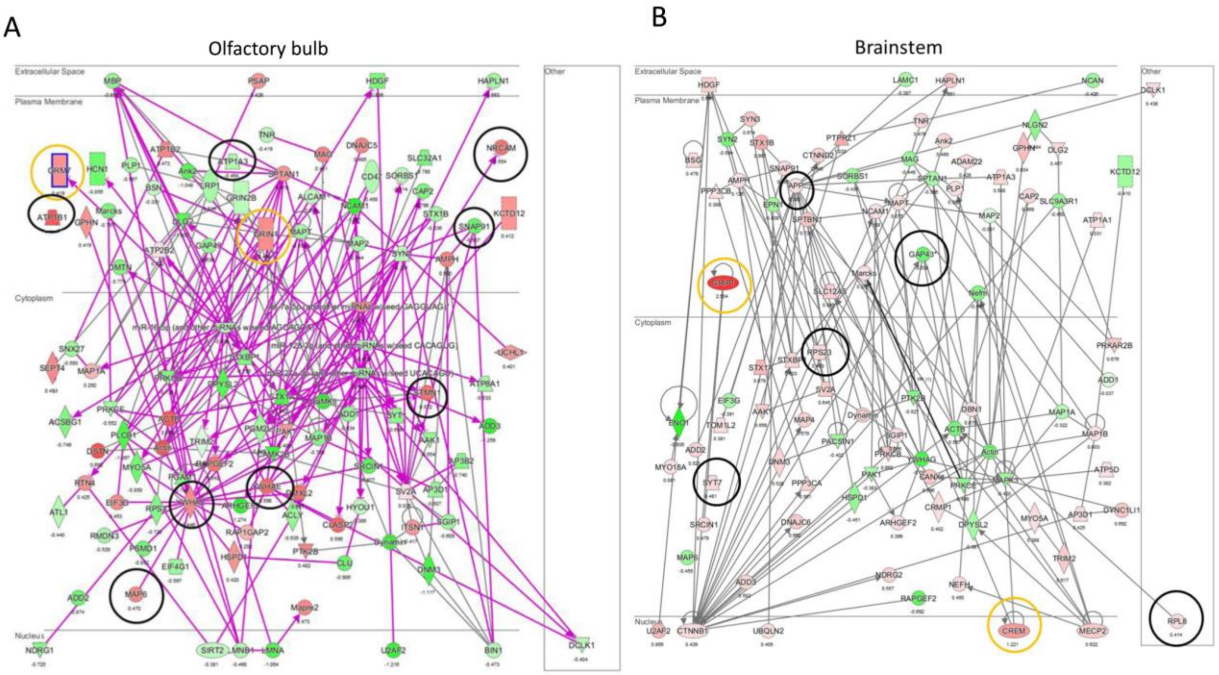

SI Figure S3: Molecular network including miRNA, mRNA, proteins and modified proteins

The figure shows the integrative networks in olfactory bulb (A) and brainstem (B) with the significant changed data of miRNA, mRNA and protein (non-modified, phospho- and N-linked glycosylated-proteins). Black circles: non-modified protein; orange circle: mRNA transcript; no circle: phospho-/glyco-protein or miRNA as indicated below; hit in red, up-regulated expression; hit in green, down-regulated expression. The colorcode of the arrows is irrevelant.
